# Validation of salt intake measurements: comparisons of a food record checklist and spot-urine collection to 24-h urine collection

**DOI:** 10.1017/S1368980022001537

**Published:** 2022-07-19

**Authors:** Sigrid Beer-Borst, Stefanie Hayoz, Corinna Gréa Krause, Pasquale Strazzullo

**Affiliations:** 1 University of Bern, Institute of Social and Preventive Medicine, Mittelstrasse 43, 3012 Bern, Switzerland; 2 Federico II University of Naples Medical School, Department of Clinical Medicine & Surgery, Naples, Italy

**Keywords:** Na, Salt intake monitoring, Validation study, Questionnaire assessment, Food record checklist, Spot-urine, 24-h urine, Prediction model

## Abstract

**Objective:**

Monitoring population salt intake is operationally and economically challenging. We explored whether a questionnaire assessment and a prediction of Na intake from spot-urine could replace or complement the recommended measurement of Na in 24-h urine (24-h U).

**Design:**

Compare the agreement of a Na-specific food record checklist (FRCL) and a late-afternoon spot-urine measurement (PM-spot) with 24-h U measurement in estimating Na intake at group level. Each participant’s use of these methods extended over 3 d. Agreement was assessed using mean (95 % CI) differences, linear regression models and Bland−Altman plots.

**Setting:**

The validation study was part of a 1-year workplace intervention trial to lower salt intake in Switzerland.

**Participants:**

Seventy women and 71 men, aged 21−61 years, completed three FRCL, and acceptable PM-spot and 24-h U samples at baseline (April−October 2015).

**Results:**

Mean Na intake estimates varied slightly across methods (3·5-3·9 g/d). Mean Na intake differences from 24-h U were 0·2 (95% CI (0, 0·5)) g/d for FRCL and 0·4 (95 % CI (0·2, 0·6)) g/d for PM-spot. Linear regression models and Bland-Altmann plots more clearly depicted differences by sex and discretionary salt use.

**Conclusions:**

Although 24-h U remains the best reference method for monitoring Na intake at the population level, PM-spot and FRCL might be more practical instruments for frequent, periodic Na intake assessments. Population-specific prediction models to estimate 24-h U could be developed and evaluated.

Governments of many countries have established initiatives to reduce excessive Na or salt intake in the population^(1)^ due to its association with high blood pressure, a major CVD risk factor^(2,3)^. When developing a national salt reduction strategy^(4)^, the Swiss government relied on data from a national cross-sectional survey of salt intake in 2010−2011^(5)^, which used 24-h urine (24-h U) collection, the recommended standard for measuring intake of both Na and K^(6)^. The impact of salt reduction measures on mean population salt intake, in particular of reformulations of salty foods, has still to be evaluated in Switzerland^(7)^. However, regular nationwide surveys of 24-h urinary Na − and ideally K, iodine and creatinine excretions as well − are costly and potentially of low reach due to a perceived high burden in collecting adequate 24-h U specimens. Other countries face the same challenges as periodically discussed within the WHO Action Network on Salt Reduction in the Population in the European Region (ESAN)^(8)^.

One popular alternative to 24-h U measurement is estimating Na intake from spot-urine collections, which have known specificities and limitations^(9–14)^. Different approaches exist as to time of collection, and different population-specific estimating equations have been developed to take into account ethnicity, sex, age, and body weight and height^(12,15–18)^. Another alternative is dietary assessment methods such as 24-h dietary recall, FFQ and food record, though these are not considered accurate enough for estimating Na intake^(19–22)^. Still, the WHO/PAHO recommends combining 24-h U collection with food intake information to identify the major Na intake sources^(6,9)^.

In the search for alternative, practical and scientifically acceptable approaches to the measurement of Na/salt intake, we built on results of an earlier study conducted in Switzerland^(23,24)^. That study assessed mean Na intake via an FFQ in a Swiss regional population and calibrated the FFQ-estimated Na intake using 24-h urinary Na measured in a subsample of the study population; its results matched mean population intakes of the Swiss salt survey^(5)^. In this new, 1-year salt intake intervention trial, we evaluated the relative validity of salt intake estimates assessed with a newly established Na- and K-specific food record checklist (FRCL) and based on a late-afternoon spoturine collection (PM-spot) by comparing both to the estimate from a 24-h U collection (reference method). This article focuses on only Na.

## Methods

### Setting and participants

The validation study was part of a 1-year workplace intervention trial to lower salt intake in Switzerland approved by Swissethics (KEK BE 130/14, PB_2016_01156) and registered in the German Clinical Trials Register (DRKS00006790, 23/09/2014). In a population of 145 consenting employees, we collected baseline data from 141 individuals of 21−61 years old, 70 women and 71 men, from April to October 2015 prior to the start of the intervention programme based on predetermined exclusion criteria (see below). Details of the intervention study protocol, particularly of the design, data collection methods and participants’ baseline characteristics are published elsewhere^(25,26)^.

**Fig. 1:**
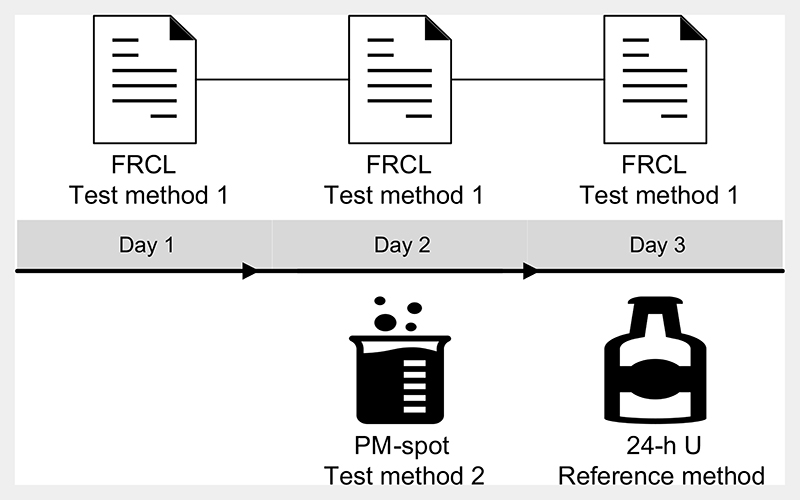
Three-day measurement protocol to estimate Na intake in the study population. Flowchart of the combination of methods across 3 d: the food record checklist (FRCL) completed on days 1 to 3 for calculation of the mean, test method 1; the late-afternoon spot-urine (PM-spot) collected on day 2, test method 2; the 24-h urine (24-h U) collected on day 3, reference method

We relied on a 3-d measurement protocol that combined three methods to estimate Na intake of the same participants: two test methods, a FRCL and a PM-spot, and one reference method, a 24-h U collection (Fig. 1). The protocol was set up to address several issues. First, the Na level in 24-h U must reflect salt intake over the same time period as the dietary questionnaire method. Second, spot-urine collection should be based on a non-fasting casual urine sample and be collected on a different day than the 24-h U in order to: (a) mimic an everyday situation applicable in a population-based salt intake monitoring system; (b) conform to the study protocol for development of the applied prediction model^(12)^; and (c) minimise the possibility of a spurious association due to day-specific factors of biological nature. Finally, the predicted 24-h Na level from spoturine and the 24-h U Na excretion must reflect the salt intake over the same time period, and factors potentially affecting Na concentration in spot-urine must be controlled.

The trained research staff instructed every participant in person about the procedures and use of the tools during a previously agreed upon 3-d period. Participants received additional written instructions and the labelled materials, which study staff picked up and checked for completeness on day 4. The urine was analysed by the Dr Risch medical laboratory, in collaboration with the laboratories of the Food Safety and Veterinary Office (FSVO), in Bern, Switzerland^(25)^. Study data were collected and managed using REDCap electronic data capture tools hosted at the Clinical Trials Unit, University of Bern^(27)^.

### Food record checklist (FRCL), test method 1

Study participants completed the semiquantitative FRCL^(28)^ on three consecutive days (day 1 to 3) irrespective of the day of the week (Fig. 1).

The questionnaire was developed to identify the main Na- and K-containing food sources eaten throughout the day by consumption frequency and amount. The tool was thus constructed as a hybrid of an FFQ and a food record for convenient self-administration at time of consumption (paper and pencil). Relying on published data^(6,23,29–40)^, a comprehensive, closed-ended list of Na- and K-containing food sources in the Swiss diet was compiled and then arranged by thirteen food categories and by thirty-five subgroups of similar foods with comparable Na/K content with examples therein. The food categories were sorted so that they reflect the sequence of customary consumption occasions. Food additions such as spreads, sauces and dressings were added to some subgroups. Aiming at an approximate estimate of the amount of food consumed, we assigned a practical reference portion to each subgroup based on published information^(41–44)^, standard sales quantity units or portion size recommendations on food labels. A grid guided recording over the course of the day: the user ticked the total number of reference portions (1 to 5+) of a food eaten at a specific food consumption occasion (breakfast, AM snack, lunch, PM snack, dinner and late hour meal). The use of discretionary salt was qualitatively assessed by a separate question. For each of the six food consumption occasions, the user ticked whether he/she had used salt and salt-containing condiments or not. The FRCL was complemented with general questions on food choice in the staff canteen. For structure and details of the FRCL, including written instructions for use, see questionnaire^(28)^.

For calculation of Na and K intake by food subgroup, we compiled a database for portion size and nutrient values per subgroup and add-on items of a food category for the questionnaire. A specific weight (g) or volume (ml) was assigned to each food subgroup reference portion and add-on item considering the mean value of a referenced weight range, or the weight of a midsize portion or of the typical Swiss product unit. For compilation of the nutrient information (mg Na or K/100 g), we relied on the Swiss Food Composition Database (SFCD V5.1)^(40)^. In cases of missing data, we consulted laboratory data from own studies^(45,46)^ or other food composition tables^(47,48)^. We generally applied the median Na (K) content of the listed single, ready-to-eat products associated with the FRCL subgroup. But if the SFCD also provided a group Na (K) value, we chose the higher of the values.

The development process was accompanied by iterative testing. First, an internal face validity test was conducted to improve the overall questionnaire format and food list completeness. The extended research group (*n* 10), which included non-nutritionists and individuals eating mixed or vegetarian diets, completed the checklist on working and weekend days. We adapted the instrument based on structured written feedback. The new version underwent a standard pre-test in everyday life. A group of twenty-six students completed an FRCL, on different days of the week, accompanied by a feedback form. They evaluated ease of use of the FRCL given instructions and examples; the closed-ended list of foods confused some persons. Reference portions were mostly considered adequate. Median daily Na (3·5 g) and K (2·6 g) intakes were in the range of population intake data from earlier salt studies in Switzerland^(5,23)^. Daily Na (salt) intakes above 3·94 g (10 g) (*n* 8) could be explained by food selection and reporting mistakes, or may be related to defined reference portion sizes. To reduce the potential risk of unrecorded or falsely allocated foods, more specific food examples were added to selected subgroups, and the format of the add-on option was simplified.

We used the resulting FRCL^(28)^ in the intervention trial. The trained research staff explained how to use the tool and interpret the closed-ended food list to every participant. To determine individual daily Na intake, we calculated the mean daily intake from the FRCL data from three consecutive days and by food group. Discretionary salt use was indicated by the use of salt or salt-containing condiments on at least one of 3 d.

We hypothesised that the FRCL produces a valid estimate of population Na intake via food. Although discretionary salt use was not quantitatively assessed, we used the qualitative information (use v. no use as described above) to refine the evaluation of Na intake assessed using the FRCL by subgroup analysis. We thus could estimate approximate Na intake from discretionary salt using linear regression analysis. We assumed that over- and underreporting by participants balance out at the group level.

### Late-afternoon spot-urine collection (PM-spot), test method 2

Study participants collected a midstream PM-spot urine void in a 100-ml container on the second day (day 2) (Fig. 1).

We based the choice of a late-afternoon spot-urine sample on findings that the correlation coefficient between 24-h and spot urinary Na excretion was better for samples collected in the late-afternoon/early evening than samples obtained before noon^(17)^. This is probably traceable to the urine volume (a factor influencing urinary Na concentration) being more stable in the afternoon rather than in the morning hours, which also makes Na concentration fairly stable at the same time^(49)^. Collection was thus scheduled for around 17 h (5 PM), which is at the midpoint of a typical morning-to-morning 24-h period well after lunch and before dinner. In addition participants completed a spot-urine recording sheet, reporting date and actual time of voiding urine, the time and kind of last food intake, the kind and intensity of physical activities during the previous 4 h, and medication use during the day^(25)^. In this way, factors potentially interfering in the prediction of 24-h Na excretion and the population estimate of daily salt intake from spot-urine could be accounted for. Apart from an individual’s day-to-day and hour-to-hour variation of Na intake, recent fluid intake, protein-rich meals and intense physical exercise may all affect the glomerular filtration rate and tubular Na handling, eventually affecting Na excretion. The concomitant measurement of urinary creatinine was supposed to, at least partially, enable accounting for these potential confounders.

For the prediction of 24-h urinary Na excretion, we chose the Danish model by Toft *et al*.^(12)^. This model has been shown to be valid, and more accurate and precise on a population level than the Tanaka equation, and is suitable for monitoring salt intake in a Swiss population differentiated by sex. The Danish model is based on the measurement of Na and creatinine concentrations in a casual spot-urine sample collected throughout the day and does not rely on 24-h urinary creatinine reference values. 24-h U and spot-urine were collected on different days. Table 1 shows the prediction equations for men and women^(12)^. Body weight and height were measured to the nearest 0·1 kg and 0·1 cm in light clothing and without shoes at the time of individual participant instruction^(25)^. Spot-urine Na and spot-urine creatinine concentrations were measured with the same laboratory methods as were used for 24-h U (see below).

We hypothesised that the PM-spot measurement and the Toft *et al*.^(12)^ prediction model produce a valid group Na intake estimate and thus will be suitable for observing Na/salt intake trends at population level.

### 24-h urine (24-h U) collection, reference method

24-h U mainly reflects recent nutrient intake over 2 d prior to and on the day of collection^(50)^. The study participants began collection of their 24-h U on the third day after discarding the first morning urine (at a time recorded as the start time) (Fig. 1). Following the WHO/PAHO standard protocol^(6)^, they then collected in 2-litre and 3-litre bottles all urine voided until the next morning, ending collection at approximately the same hour as the start time (recorded as the finish time). Participants noted relevant information about date and time, spoiled or missed urine collections, and medication use on the 24-h U collection recording sheets we provided.

The collection of 24-h U samples is prone to error. We accepted collection durations of 24 h +/- 4 h (20−28 h) but excluded urine samples if less than 300-ml urine had been collected, two or more voidings were reported missing, or urinary creatinine excretion was above 400 μmol/kg/24 h^(25,51)^. Quality of acceptable urine samples was further evaluated by comparing 24-h creatinine excretion with Swiss 24-h creatinine reference values^(52)^. Those with inadequate creatinine values underwent a plausibility check assessing explanatory information and a sensitivity test to determine inclusion in the study^(25)^. The 24-h U samples of 141 study participants met criteria for inclusion in the trial baseline and validation analysis.

### Laboratory urine analysis and estimation of Na/salt intake from urinary excretions

Na concentrations (mmol/l) in PM-spot and 24-h U aliquots were analysed using an indirect ion-selective electrode technique (Roche eLabDoc ISE indirect Na-K-Cl for Gen.2 Global, V9.0). In cases of Na concentrations below the detection limit of 20 mmol/l, a retained aliquot was analysed with the more sensitive ICP-OES method^(25)^. Creatinine concentrations (μmol/l) were quantified with the kinetic Jaffe method (Roche eLabDoc Creatinin Jaffé Gen.2, V17.1)^(25)^.

The volumes of the individual 24-h U collections were gravimetrically determined, and urinary measures were standardised to a 24-h period by adjusting the volume for the reported collection duration.

For comparison with Na intake assessed via the FRCL, daily Na intake (g/24 h) was estimated by multiplying predicted (spot) and measured urinary Na excretion in 24 h (mmol/24 h) by the atomic weight of Na (23 mg/mmol). The daily salt equivalent intake was derived by multiplying the Na intake estimates (g/24 h) by 2·54.

### Validation statistics

Continuous variables were summarised using median and range as well as mean and 95 % CI and graphically illustrated using boxplots. Categorical variables were summarised using frequency and percentage. In the estimation of Na intake, differences between the FRCL measurement and the PM-spot measurement, and the 24-h U measurement were calculated and summarised using mean and 95 % CI and graphically illustrated using forest plots. Bland−Altman plots and linear regression models were applied to assess agreement between FRCL and PM-spot with 24-h U measurement. Subgroup analyses were performed by sex and by discretionary salt use. All statistical analyses were performed using R version 3.4.3.

## Results

### Characteristics of participants and urine collection attributes

Table 2 shows selected characteristics of the 141 mostly well-educated participants with complete 24-h U specimens. The sexes were equally represented, and median age was 46 years. Self-reported and measured indicators suggest that overall the group presented as health conscious. Still, a non-negligible proportion of participants manifested critical blood pressure levels and weight status.

The self-reported information about 24-h U and PM-spot collections is summarised in Supplemental Tables S1 and S2. Median 24-h U collection duration was 24 h and the median time-standardised urine volume was 2·25 l/24 h (see online Supplemental Table S1). Ninety-four percent of participants collected their PM-spot within +/− 1 h of the predetermined collection time (17 h/5 PM). Fifty-seven percent of participants had their last food intake within 2 h of the PM-spot, and independent of the time lag 38 % reported having consumed protein-rich foods. Few participants (11 %) reported having performed vigorous intensity physical activity within 3−4 h of the PM-spot (see online Supplemental Table S2). During the day of 24-h U collection, 23 % of participants reported use of at least one of five potentially critical drug types, while 22 % reported such use on the day of PM-spot urine collection (see online Supplemental Tables S1 and S2). Mean creatinine and Na concentration in PM-spot were higher for participants who reported their last food intake 3 h or more prior to urine collection, and also among those who used medication (see online Supplemental Tables S3 and S4). Consumption of protein-rich foods prior to spot collection made no major difference to mean Na concentration (see online Supplemental Table S4), although it was associated with a higher mean creatinine concentration (see online Supplemental Table S3). Mean creatinine concentration in PM-spot was highest and Na concentration was lowest for the subgroup of participants who performed vigorous intensity physical activities (see online Supplemental Tables S3 and S4). Applying the prediction model (Table 1), the estimated mean daily Na (salt) intake showed the same patterns for time to most recent food intake and physical activity (see online Supplemental Tables S5a and S5b).

### Daily Na intake across methods

Overall mean Na intake estimates varied across methods from 3·5 to 3·9 g/d. Women’s mean and median daily Na intake estimates were always lower than those of men (Table 3).

Supplemental Figure S1 visualises the distributions of daily Na intake predicted from PM-spot, determined from 24-h U and assessed via the FRCL in greater detail. Overall, median PM-spot and FRCL Na intake estimates (both 3·6 g/d) were above the median 24-h U Na intake estimate (3·1 g/d), but variance looked quite similar across methods. However, stratification by sex displayed dissimilarities (Table 3 and see online Supplemental Fig. S1 panels b and c). In women, the variance was largest for the FRCL estimate, and the distribution of Na intake predicted from PM-spot urine differed markedly from the one of 24-h U Na intake measurement. In men, the variance for the FRCL estimate and for the 24-h U estimate were similar, and the FRCL Na intake estimation compared best with 24-h U.

According to the FRCL, five food groups were responsible for 80 % of mean daily Na/salt intake of women and men. According to self-reports, the use of discretionary salt, which is not accounted for in the Na/salt intake estimate via FRCL, was more frequent among women than men across four subgroups of salt intake (see online Supplemental Table S6).

### Agreement of Na intake estimations

**Fig. 2:**
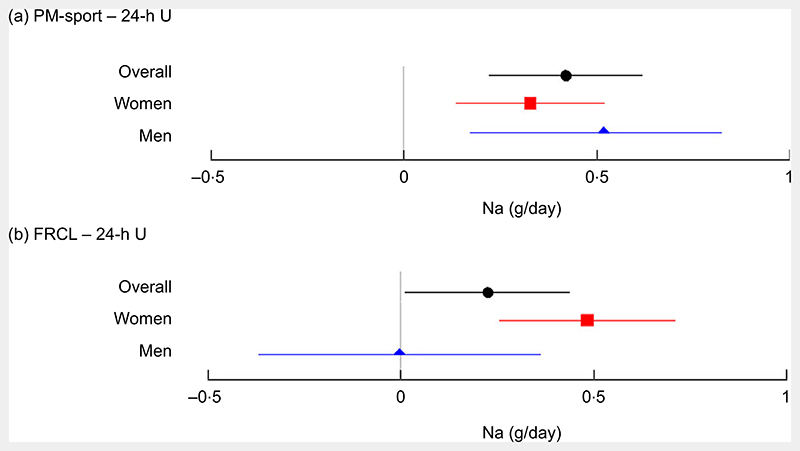
Mean (95 % CI) differences in estimated daily Na intake between each test method and the reference method, overall and by sex. Forest plots of mean differences in daily Na intake estimates from late-afternoon spot-urine excretion (PM-spot) (Toft prediction model^(12)^) and 24-h urinary excretion (24-h U) (panel a), and from assessment via food record checklist (FRCL) and 24-h U (panel b), overall (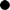 *n* 141), and for women (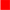 *n* 70) and men (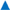 *n* 71)

The overall mean (95 % CI) differences in estimated daily Na intake from 24-h U were 0·4 (95 % CI (0·2, 0·6)) g for PM-spot and 0·2 (95 % CI (0, 0·5)) g for FRCL (Fig. 2 panels a and b).

For PM-spot, the mean differences overall and by sex were significant and indicated overestimation of actual Na intake (Fig. 2 panel a). With the FRCL, men − but not women − appeared to estimate actual Na intake quite well, but mean differences in men, and most probably also overall, were NS (Fig. 2 panel b).

**Fig. 3:**
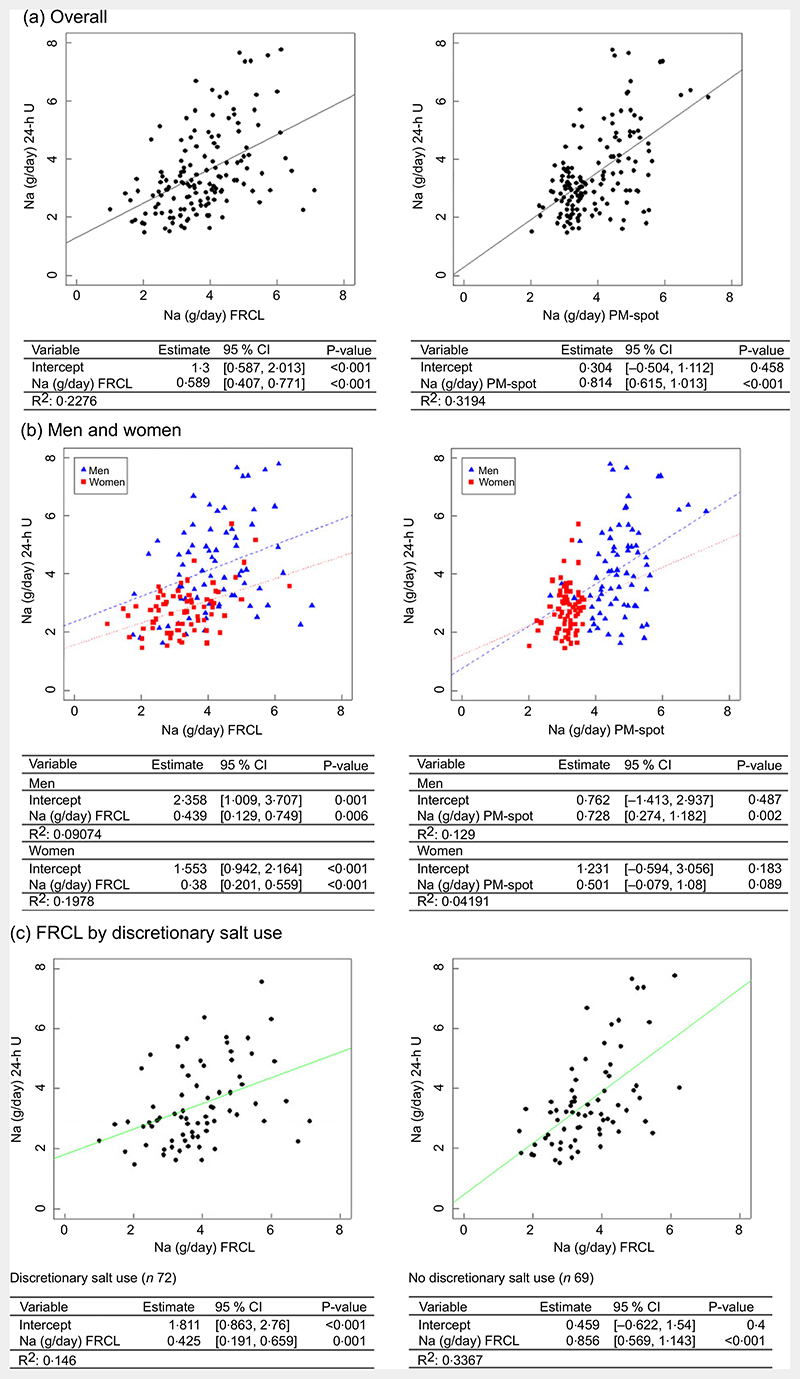
Linear regression models to predict daily Na intake from FRCL and PM-spot. Linear regression models for predicting daily Na intake as measured/estimated from 24-h urinary excretion (24-h U) from assessment via food record checklist (FRCL) and late-after-noon spot-urine excretion (PM-spot) (Toft prediction model^(12)^) overall (panel a;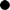 *n* 141), by sex (panel b; women 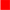 *n* 70, men 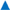 *n* 71), and (panel c) prediction from FRCL by discretionary salt use

Both overall linear regression models, for FRCL and for PM-spot (Fig. 3 panel a), did not predict the actual Na intake (24-h U reference) very well.

According to the model, individuals generally underestimated their actual Na intake when using the FRCL (intercept 1·3, *P* < 0.001), even though those with higher recorded Na intakes tended to overestimation. Prediction of actual Na intake using PM-spot produced a better fitting regression line (intercept 0·3, P = 0·5); the prediction based on PM-spot seemed to be more prone to underestimation (Fig. 3 panel a). Besides, frequencies of misclassification of Na intake estimate in adjacent or opposite tertiles of 24-h U was quite similar for FRCL (53 %) and PM-spot (49 %) (see online Supplemental Table S7).

Linear regression analysis by sex (Fig. 3 panel b) revealed that the FRCL model, though showing flat, linear slopes, fit the actual Na intake better than the PM-spot model (sex−PM−spot interaction), particularly for women.

Because a significant interaction between Na intake and reported discretionary salt use was found for the FRCL (see online Supplemental Table S8), separate models were applied for discretionary salt users and non-users. Figure 3 panel c shows that the linear regression model for the FRCL fits better for all non-users of discretionary salt. Average daily Na intake from discretionary salt, represented by the intercept, was 1·8 (95 % CI (0·863, 2·76)) g.

**Fig. 4:**
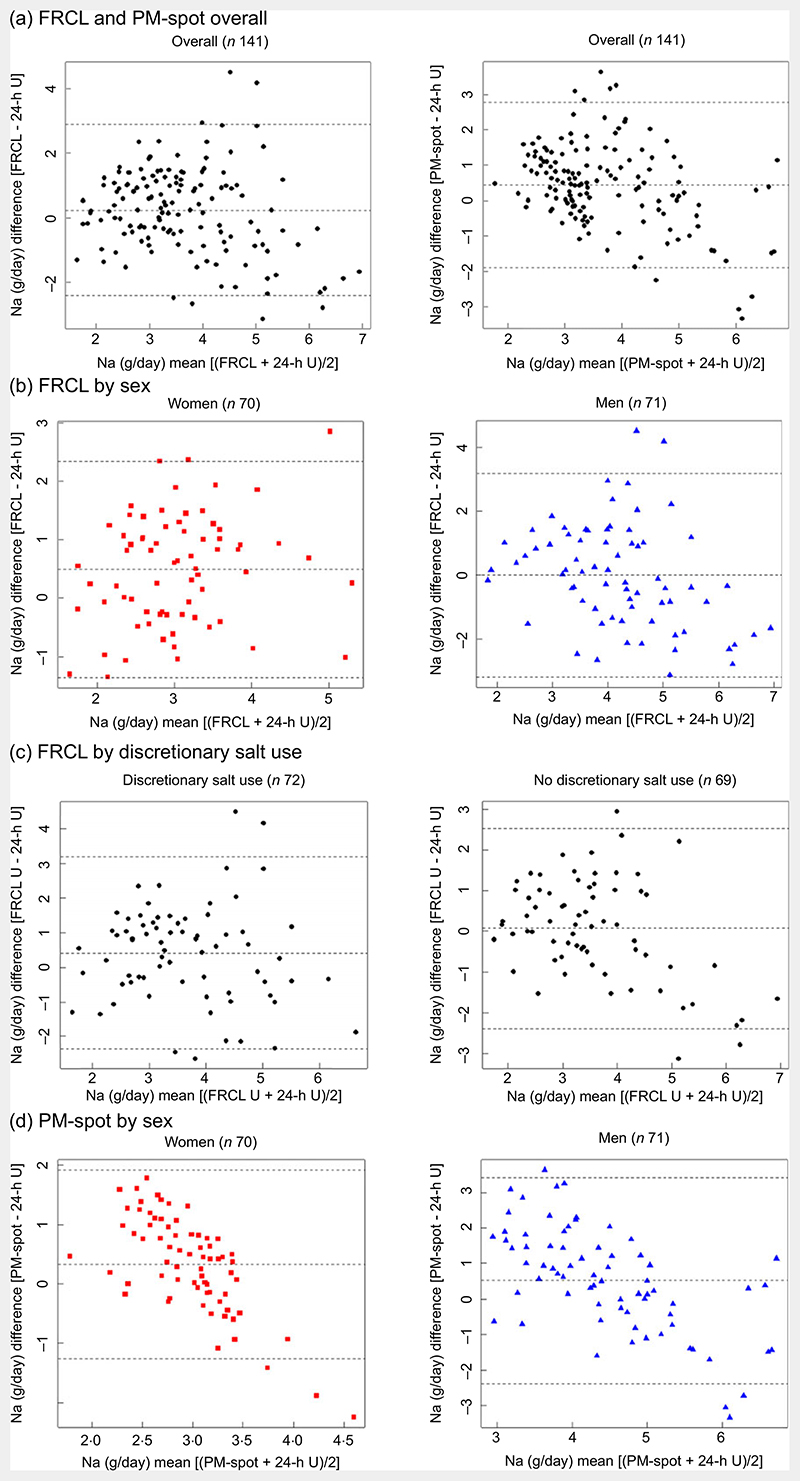
B and−Altman difference against mean plots. Agreement between each test method (food record checklist (FRCL); late-after-noon spot-urine excretion (PM-spot) (Toft prediction model^(12)^)) and the reference method (24-h urinary excretion (24-h U)) in estimating daily Na intake, overall (panel a; 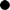 *n* 141), by sex (panels b and d; women 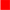 *n* 70, men 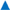 *n* 71), and (panel c) for FRCL by discretionary salt use

According to Bland−Altman analysis, the FRCL produced a less biased estimate of actual Na intake than did PM-spot (Fig. 4 panels a-d).

Overall, the mean of individual differences was closer to zero for the FRCL. However, the questionnaire provided less robust data for higher Na intakes (Fig. 4 panel a); this was particularly true for men (Fig. 4 panel b). The pictures for agreement of FRCL and 24-h U estimates by sex (Fig. 4 panel b) and by discretionary salt use (Fig. 4 panel c) were similar. The mean of individual differences was zero for non-users of discretionary salt, but the scatter showed more structure with higher Na intakes.

## Discussion

### Study protocol

This validation study was part of a 1-year intervention trial implemented under everyday conditions in the Germanspeaking population of Switzerland^(25)^. Three different methods of Na excretion or Na intake assessment were used with each person and compared for potential use in a population salt intake monitoring system. A 3-d measurement protocol (see methods, Fig. 1) evaluated the degree of agreement between each of the two test methods, FRCL and PM-spot, and the 24-h U reference method, with each measuring the same thing. Women and men were equally represented and in spite of the burdensome measurement protocol and small sample size (n 141) participation was still sufficient to conduct a validation analysis by sex, a factor shown to effect population Na/salt intake^(26,51)^. The quality of the data is good. The generally well-educated participants were closely supervised during the trial. Their FRCL and urine collection recording sheets were checked for completeness upon return, and study staff contacted participants to verify missing or inconsistent information prior to data entry^(25)^. Continuous completion of the FRCL required discipline, and urine collection remained challenging for everyone^(25,26)^. Limitations inhere in each of the three methods that are independent of the objective biological (PM-sport and 24-h U) or the subjective self-reported (FRCL) character of the measure. Within-individual variation is an issue for all three measures alike. Urinary Na is regarded as a recovery biomarker. That is, it provides an estimate of absolute Na intake over a fixed time period. However, the 24-h U method is considered burdensome and people may produce unacceptable and inadequate samples (see methods). It still serves as the best existing reference method, though, and compliance with the 24-h U collection protocol in our study was high with 141 of 145 baseline 24-h U collections acceptable and adequate. This was also supported by the self-recorded information about 24-h U collection (see online Supplemental Table S1).

Validation took a standard statistical approach. Linear regression (Fig. 3) and Bland−Altman (Fig. 4) analyses were used to look at patterns of individual measurement results overall and by subgroups, always keeping in mind the main idea that the test methods should predict group Na intake. Ideally, the reference Na intake would have been based on more than a single 24-h U. The day-to-day variability of an individual’s daily Na excretion generally reduces the strength of any possible correlation with the test methods. Still, the application of both analyses allowed a more accurate assessment of the test methods’ performance by directly comparing the Na intake estimates and mean differences between test and reference methods (Table 3 and Fig. 2).

### Main results

In general, the FRCL better agreed with the 24-h U than did the PM-spot.

#### Main results - performance of test method (1) FRCL

The development of the FRCL followed a standard protocol. The tool represents a new, Na-specific hybrid of an FFQ and a food record. Therefore, direct comparisons with results from validation studies for non-nutrient-specific FFQ or dietary records^(21,22)^ were not made here. We created a comprehensive but still manageable list of food subgroups per food category by providing several typical food examples per subgroup.

The assumption that participants’ over- and underreporting of Na intake in food will balance out at the group level was partially confirmed. The FRCL provided the best agreement at daily Na intake levels of 2·5-3·5 g in women and 3·5-4·5 g in men. The use of mean individual intakes of three consecutively applied FRCL may have reduced the random component of within-individual variation for the questionnaire measure. The generally observed underestimation of actual Na intake (Fig. 3) was expected because the FRCL does not quantify discretionary salt use. The fact that individuals with higher recorded Na intake tended to overestimate the actual intake may be mainly due to misperception of serving sizes, or the wish to report consumed but not listed foods. Regardless, the qualitative information about discretionary salt use proved crucial as a correction factor in the analysis of FRCL assessment. The Bland−Altman plots by sex and by discretionary salt use resembled one another because more women than men reported use of discretionary salt. The average amount of discretionary salt (Fig. 3 panel c, intercept 1·8 g/dNa or 4·6 g/d salt) confirms earlier estimates in Switzerland^(23)^. In general, the FRCL is expected to have the better reach than urine collection − especially 24-h U − due to wider acceptability or higher compliance. This might be particularly true if mobile applications could become an option for ensuring less burdensome recording throughout the day. This could also allow using sex-specific standard portion sizes for certain foods to gain accuracy of Na intake estimation. Also, knowing food sources of Na intake is important when implementing salt reduction strategies via food reformulation programmes and education intervention. However, the database of food Na composition requires regular updates with ongoing reformulations, and reference portion sizes may vary over time. The establishment of an FRCL-based Na intake prediction model is possible, but its use would be complicated by the need for updates.

#### Main results − performance of test method (2) PM-spot

Using PM-spot for group estimates of Na intake proved feasible, though not all participants may have collected a midstream urine void. The information on food intake and physical activities collected in conjunction with PM-spot collection proved helpful in evaluating the impact of these factors, via subgroup analysis, on creatinine (see online Supplemental Tables S3) and Na concentrations in PM-spot (see online Supplemental Tables S4), and on predicted Na (salt) intake (see online Supplemental Tables S5a and S5b). This information may also help refine the performance of the PM-spot method and prediction of Na intake. Consumption of protein-rich foods primarily affected creatinine concentration, but not Na concentration in PM-spot. It further appears that the longer the interval between the last intake of food and spot collection, the higher the concentration of both Na and creatinine in spot-urine (see online Supplemental Tables S3 and S4), which is probably due to higher absorption. By contrast, physical exercise 3−4 h prior to urine collection lowered Na concentration in spot-urine (see online Supplemental Table S4). This was possibly due to Na loss via intense perspiration. Higher creatinine concentration (see online Supplemental Table S3) attributable to higher muscular mass and muscle strain may have produced lower predicted Na intake values for physically active people (see online Supplemental Table S5a). In addition, any food intake of highly active persons before spot collection did not seem to result in a higher Na intake measurement. The interval between food intake and spot collection may have been short, and athletes may be more health conscious and have lower salt consumption habits. The observations must be interpreted with care, though, in view of the small size (*n* 15) of the subgroup.

The Danish prediction model^(12)^ provided generally reasonable results, but the formula for women appeared less suitable than the one for men (see online Supplemental Fig. S1 and Fig. 3, panels b; Fig. 4 panel d). Possible factors in this may include the random error associated with biological variation of urinary Na excretion within and between the (rather few) female participants. It thus would be desirable to verify this observation by repeating the validation study with at least two individual PM-spot urine collections in a larger population sample of women and men. Because our study was restricted to the German-speaking part of Switzerland, but patterns of food intake differ across Swiss linguistic regions^(53)^, a nationwide sample should be selected. Moreover, information about the performance of the prediction model^(12)^ in other populations, and particularly in women, would be helpful. If no satisfactory results are obtained, an appropriate Swiss prediction model for 24-h Na and K excretion from spot-urine should be subsequently established to better reflect population specifics.

As expected, both test methods had their limitations and a replacement of 24-h U by either the FRCL or PM-spot does not seem apt. Neither provides reliable information at the individual level, though this is also true for a single 24-h U collection.

We assume that the results would be similar when methods are applied in different countries provided the FRCL is first adapted to country-specific food markets and intake patterns.

## Conclusion

Switzerland cannot yet build on any regularly repeated measurements of national salt intake; this may be true for many other countries as well. Collecting information about population salt intake requires a pragmatic approach. Although the 24-h U remains the best reference method for monitoring Na intake at the population level, PM-spot collection is clearly less burdensome than 24-h U collection, and the FRCL may provide additional important information about sources of Na and K in food. The PM-spot and FRCL might thus be more practical instruments for intake assessments in a system for monitoring dietary risk factors. No matter whether the prediction model of Toft *et al.*^(12)^ or a newly developed Swiss prediction model is implemented nationally, we suggest using a combination of the test and 24-h U reference methods (see online Supplemental Fig. S2), and regular recalibration of the test methods to obtain reliable information on changing salt intake. Ideally, a statistically representative national nutrition cohort of adequate size of women and men 15 years of age and older would yield necessary data. Organisation and long-term funding must be ensured as part of the implementation of nutrition policy.

## Supplementary Material

online supplemental tables and figures

## Figures and Tables

**Table 1:** Sex-specific equations of the Danish prediction model^(12)^

	Men	Women
PRCr, predicted 24-h creatinine excretion (mg/d)	−7·54 age (years) + 14·15 weight (kg) + 3·48 height (cm) + 423·15	−6·13 age (years) + 9·97 weight (kg) + 2·45 height (cm) + 342·73
XNa (mmol/d)	SUNa (mmol/l)/SUCr (mg/l) × PRCr (mg/d)	SUNa (mmol/l)/SUCr (mg/l) × PRCr (mg/d)
PRNa, predicted 24-h Na excretion (mmol/d)	33·56 × XNa^0·345^	52·65 × XNa^0·196^

SUNa, spot-urine Na; SUCr, spot-urine creatinine.

**Table 2:** Characteristics of the validation study participants^(25,26)^

Number participants	*n*	%
Total	141	
Women	70	49·6
Men	71	50·4
**Age (years)**	Median	Range
	46	21−61
**Education, tertiary level**	*n*	%
	104	73·8
**Salt awareness (self-reported)**	*n*	%
No discretionary salt use	66	46·8
Know recommended salt intake	73	51·8
Salt content impacts food/menu choice	61	43·3
**Smoking, never and former**	*n*	%
	119	84·4
**Physical activity**		
Meeting WHO recommendations for health^*^	*n*	%
	134	95
Daily time sitting (min/d)	Mean	95 % CI
	450·9	418·7, 483·0
**Blood pressure (mmHg)**	Mean	95 % CI
Systolic	119·8	117·4, 122·2
Diastolic	73·2	71·5, 74·9
Hypertension^†^	n	%
	22	15·6
**Body composition**	Median	Range
Weight (kg)	72·6	47·6−114·7
Height (cm)	173·4	150·9−193·5
BMI (kg/m^2^)	Mean	95 % CI
	24·6	24·0, 25·3
	n	%
Overweight or obese, BMI? 25 kg/m^2^	57	40·4
Waist-to-height ratio (WHtR)? 0·5^‡^	70	49·6

* According to Global Physical Activity Questionnaire (GPAQ) (WHO), 150-min moderate intensity PA or 75-min vigorous intensity PA, or equivalent combination achieving 600 + MET/week.

† Systolic blood pressure (BP) ≥ 140 mmHg and/or diastolic BP ≥ 90 mmHg and/or current intake of BP lowering drugs.

‡ WHtR ≥ 0·5 indicates an increased risk for CVD and diabetes.

**Table 3:** Mean (95% CI) and median (range) daily Na intake estimates across methods, overall and by sex

	Na intake estimates (g/d)							
Methods	PM-spot		24-h U		FRCL	
**Overall (*n* 141)**						
Mean (95 % CI)	3·9	3·8, 4·1	3·5	3·3, 3·7	3·7	3·6, 3·9
Median (range)	3·6	2·0−7·3	3·1	1·5−7·8	3·6	1·0−7·1
**Women (*n* 70)**						
Mean (95 % CI)	3·1	3·1, 3·2	2·8	2·6, 3·0	3·3	3·0, 3·5
Median (range)	3·2	2·0−3·6	2·8	1·5−5·7	3·2	1·0−6·4
**Men (*n* 71)**						
Mean (95 % CI)	4·7	4·5, 4·9	4·2	3·8, 4·6	4·2	3·9, 4·5
Median (range)	4·7	2·6−7·3	4·0	1·6−7·8	4·1	1·8−7·1

PM-spot, intake predicted from excretion in late-afternoon spot-urine: prediction model by Toft eřaí^(12)^; 24-h U, intake calculated from excretion in 24-h urine (reference method); FRCL, food record checklist: questionnaire assessment of intake.
