# Supplementary material for: Validation of salt intake measurements: comparisons of a food record checklist and spot-urine collection to 24-h urine collection
Source: Public Health Nutr. 2022 Jul 19;25(11):2983–94. doi: 10.1017/S1368980022001537 (PMC7613718; doi:10.1017/S1368980022001537)
Supplement: Supplementary file 1 [file S1368980022001537sup001.pdf]

Validation of salt intake measurements: comparisons of a food record checklist and spot-urine collection to 24-hour-urine collection

Sigrid Beer-Borst[1], Stefanie Hayoz[1], Corinna Gréa Krause[1], Pasquale Strazzullo[2]

[1] University of Bern, Institute of Social and Preventive Medicine, Mittelstrasse 43, 3012 Bern, Switzerland.

[2] Federico II University of Naples Medical School, Department of Clinical Medicine & Surgery, via S. Pansini 5, 80131 Naples, Italy

---

SUPPLEMENTARY MATERIAL

TABLES S1 to S8

FIGURE S1 and S2

V1.2, 25.05.2022

Supplementary Table S1 Attributes of 24-hour urinary collections/samples included in the study (N=141)

**24-hour urine collection**		**N=141**
Duration (min)	Median (range)<br>Mean (95% CI)	1440 (1215 , 1590)<br>1433.4 (1426.3 , 1440.5)
Total volume (ml)	Median (range)<br>Mean (95% CI)	2235 (449 , 5063)<br>2364.4 (2207.9 , 2520.9)
Volume in 24 hours (ml/24h)	Median (range)<br>Mean (95% CI)	2249 (449 , 5063)<br>2374.1 (2217.7 , 2530.6)
Creatinine excretion in 24 hours (µmol/kg/24h)	Median (range)<br>Mean (95% CI)	178 (87.4 , 316.4)<br>184.3 (177.4 , 191.2)
**Medication use**		**N=141**
Antihypertensives (nondiuretic)	n (%)	8 (5.7%)
Diuretics	n (%)	2 (1.4%)
Anti-inflammatory drugs or painkillers	n (%)	10 (7.1%)
Thyroid hormone replacement	n (%)	4 (2.8%)
Contraceptive or hormone-replacement therapy	n (%)	10 (7.1%)

Supplementary Table S2 Attributes of PM-spot urinary collections/samples included in the study (N=141)

**PM-spot collection**		**N=141**
Time lag to 17h00 (5PM) (min)	Median (range)	5 (-150 , 180)
	Mean (sd)	11.8 (33.1)
Time lag to last food intake (min)	Median (range)	111.5 (5 , 330)
	Mean (sd)	132.3 (93.9)
**Kind of last food intake***		**N=141**
Meal (without protein component)	n (%)	51 (36.2%)
Protein-rich meal or snack	n (%)	54 (38.3%)
Snack	n (%)	54 (38.3%)
Drink	n (%)	111 (78.7%)
**Medication use**		**N=141**
Antihypertensives (nondiuretic)	n (%)	10 (7.1%)
Diuretics	n (%)	2 (1.4%)
Anti-inflammatory drugs or painkillers	n (%)	9 (6.4%)
Thyroid hormone replacement	n (%)	4 (2.8%)
Contraceptive or hormone-replacement therapy	n (%)	7 (5.0%)
**Physical activities 3-4 hours before†**		**N=141**
Low intensity	n (%)	72 (50.1%)
Moderate intensity	n (%)	54 (38.3%)
Vigorous intensity	n (%)	15 (10.6%)

* A person could have consumed different kind of foods. Meal: indication of lunch or dinner and listing of nutrient-dense foods from different food groups; classified as protein-rich meal if mention of at least one protein meal component. Snack: indication of an eating event between main meals and consumption of a single, mostly energy-dense food; classified as protein-rich snack if mention of a specific protein food source. Drink: all cold or warm liquid foods produced/prepared for drinking and to cover daily fluid requirements, does not include milk and milk-based liquids

† Multiple mentions were possible. Low intensity: mainly sitting work activities and commuting sitting in car/public transport; moderate intensity: manual work activities and human powered commuting or commuting sitting in car/public transport; vigorous intensity: sport

Supplementary Table S3 Distribution of creatinine concentration in PM-spot by attributes of spot urinary collections (N=141)

PM-spot collection attributes		n	Creatinine concentration in PM-spot (µmol/L) Min	10%	25%	50%	75%	90%	Max	Mean (sd)
**Time lag to last food intake**	≤ 60 min	39	1793.0	2126.4	3203.5	7242.0	10469.0	13106.6	18553.0	7210.8 (4557.0)
	61-180 min	41	1269.0	1674.0	2274.0	5636.0	10066.0	15251.0	24712.0	7034.1 (5908.6)
	≥ 181 min	43	897.0	2123.0	3416.0	8319.0	13517.0	15694.6	49986.0	9426.0 (8290.2)
**Kind of last food intake***	Protein-rich meal or snack	54	897.0	1775.0	3102.2	7106.0	12564.0	16155.7	49986.0	8893.5 (8187.8)
	All other kind	86	1269.0	2013.5	3111.2	6871.0	10856.8	13989.5	21362.0	7302.2 (4760.7)
**Medication use (five drug types)**	Yes	31	1143.0	2055.0	3058.5	6517.0	14202.0	16902.0	49986.0	9825.5 (9388.7)
	No	110	897.0	1945.1	3121.8	6983.5	11003.0	13815.8	24712.0	7505.6 (5105.4)
**Physical activities 3-4 hours before†**	Low intensity	72	1090.0	2064.4	3097.2	6115.5	10240.2	13255.4	24708.0	7025.7 (4683.7)
	Moderate intensity	54	897.0	1784.9	3102.2	7968.5	12309.2	15184.9	49986.0	8697.1 (7728.7)
	Vigorous intensity	15	1143.0	1537.4	3483.5	8533.0	15063.5	17669.8	24712.0	9280.4 (7193.1)

*Meal: indication of lunch or dinner and listing of nutrient-dense foods from different food groups; classified as protein-rich meal if mention of at least one protein meal component. Snack: indication of an eating event between main meals and consumption of a single, mostly energy-dense food; classified as protein-rich snack if mention of a specific protein food source

† Multiple mentions were possible. Low intensity: mainly sitting work activities and commuting sitting in car/public transport; moderate intensity: manual work activities and human powered commuting or commuting sitting in car/public transport; vigorous intensity: sport

Supplementary Table S4 Distribution of Na concentration in PM-spot by attributes of spot urinary collections (N=141)

**PM-spot collection attributes**		**n**	**Na concentration in PM-spot (mmol/L)**							
			**Min**	**10%**	**25%**	**50%**	**75%**	**90%**	**Max**	**Mean (sd)**
**Time lag to last food intake**	≤ 60 min	39	4.2	21	35.5	81	103	143.6	250	78.4 (51.7)
	61-180 min	41	13.6	25	36	86	126	167	198	89.3 (54.7)
	≥ 181 min	43	5.6	25.8	39.5	89	155	183.4	274	100.3 (65.3)
**Kind of last food intake***	Protein-rich meal or snack	54	5.6	24	34.2	85.5	130.5	167.7	206	89.5 (57.6)
	All other kind	86	2.3	24	38	83	127.5	170.5	274	89.2 (58.8)
**Medication use (five drug types)**	Yes	31	5.6	24	44.5	86	128	181	206	91.4 (56.4)
	No	110	2.3	23.9	35.2	82.5	127.5	167	274	88.5 (58.6)
**Physical activities 3-4 hours before**[†]	Low intensity	72	13.6	24.1	34.8	87	119	162.7	206	87.9 (52.3)
	Moderate intensity	54	2.3	16.8	37.2	85.5	142	176.7	274	93.8 (67.2)
	Vigorous intensity	15	24	32.4	36.5	66	107	138.4	189	78.3 (49.4)

*Meal: indication of lunch or dinner and listing of nutrient-dense foods from different food groups; classified as protein-rich meal if mention of at least one protein meal component. Snack: indication of an eating event between main meals and consumption of a single, mostly energy-dense food; classified as protein-rich snack if mention of a specific protein food source

† Multiple mentions were possible. Low intensity: mainly sitting work activities and commuting sitting in car/public transport; moderate intensity: manual work activities and human powered commuting or commuting sitting in car/public transport; vigorous intensity: sport

Supplementary Table S5a Distribution of daily Na intake estimates by attributes of PM-spot urinary collections (N=141)

PM-spot collection attributes		n	Na intake estimate (g/day)*							
			Min	10%	25%	50%	75%	90%	Max	Mean (sd)
**Time lag to last food intake**	≤ 60 min	39	2.3	2.9	3.1	3.4	4.4	4.7	5.5	3.7 (0.8)
	61-180 min	41	2.6	3.2	3.4	3.9	4.7	5.2	6.8	4.1 (1.0)
	≥ 181 min	43	2.2	2.9	3.1	3.5	5.0	5.5	7.3	4.0 (1.1)
**Kind of last food intake**[†]	Protein-rich meal or snack	54	2.2	2.9	3.1	3.5	4.5	5.2	7.3	3.8 (1.0)
	All other kind	86	2.0	3.0	3.2	3.9	4.7	5.2	6.8	4.0 (1.0)
**Medication use (five drug types)**	Yes	31	2.2	2.9	3.1	3.5	4.3	5	7.3	3.8 (1.0)
	No	110	2.0	2.9	3.1	3.7	4.7	5.3	6.8	4.0 (1.0)
**Physical activities 3-4 hours before**[‡]	Low intensity	72	2.6	3.0	3.2	4.0	4.8	5.4	7.3	4.1 (1.0)
	Moderate intensity	54	2.0	2.9	3.1	3.5	4.7	5.1	5.9	3.8 (1.0)
	Vigorous intensity	15	2.6	2.7	3.0	3.5	4.1	4.7	5.3	3.6 (0.8)

* Prediction model by Toft et al. [(12)]

[†] Meal: indication of lunch or dinner and listing of nutrient-dense foods from different food groups; classified as protein-rich meal if mention of at least one protein meal component. Snack: indication of an eating event between main meals and consumption of a single, mostly energy-dense food; classified as protein-rich snack if mention of a specific protein food source

[‡] Multiple mentions were possible. Low intensity: mainly sitting work activities and commuting sitting in car/public transport; moderate intensity: manual work activities and human powered commuting or commuting sitting in car/public transport; vigorous intensity: sport

Supplementary Table S5b Distribution of daily salt intake estimates by attributes of PM-spot urinary collections (N=141)

**PM-spot collection attributes**		**n**	**Salt intake estimate (g/day)***							
			**Min**	**10%**	**25%**	**50%**	**75%**	**90%**	**Max**	**Mean (sd)**
**Time lag to last food intake**	≤ 60 min	39	5.8	7.4	7.9	8.7	11.1	12.1	13.9	9.3 (2.0)
	61-180 min	41	6.7	8.0	8.7	10.0	11.9	13.2	17.2	10.5 (2.4)
	≥ 181 min	43	5.7	7.5	7.8	9.0	12.6	14.0	18.6	10.3 (2.9)
**Kind of last food intake**[†]	Protein-rich meal or snack	54	5.7	7.4	7.9	8.9	11.5	13.2	18.6	9.8 (2.6)
	All other kind	86	5.1	7.6	8.0	9.9	12.0	13.3	17.2	10.2 (2.5)
**Medication use (five drug types)**	Yes	31	5.7	7.4	8.0	8.8	11.0	12.6	18.6	9.6 (2.6)
	No	110	5.1	7.5	8.0	9.5	12.0	13.5	17.2	10.1 (2.5)
**Physical activities 3-4 hours before**[‡]	Low intensity	72	6.6	7.7	8.2	10.1	12.1	13.7	18.6	10.4 (2.6)
	Moderate intensity	54	5.1	7.5	7.9	8.8	11.9	13.1	15.1	9.6 (2.5)
	Vigorous intensity	15	6.6	6.8	7.6	8.9	10.4	12.0	13.5	9.1 (2.1)

* Prediction model by Toft et al. [12]

[†] Meal: indication of lunch or dinner and listing of nutrient-dense foods from different food groups; classified as protein-rich meal if mention of at least one protein meal component. Snack: indication of an eating event between main-meals and consumption of a single, mostly energy-dense food; classified as protein-rich snack if mention of a specific protein food source

[‡] Multiple mentions were possible. Low intensity: mainly sitting work activities and commuting sitting in car/public transport; moderate intensity: manual work activities and human powered commuting or commuting sitting in car/public transport; vigorous intensity: sport

Supplementary Table S6 Food group sources of daily Na/salt intake by sex and use of discretionary salt, assessed via Food record checklist (FRCL) (N=141)

**Food groups in decreasing order for all**	**Mean intake estimates***							
	**Women (n=70)**				**Men (n=71)**			
	**Na g/day**	**Salt g/day**	**%**	**Cum%**	**Na g/day**	**Salt g/day**	**%**	**Cum%**
Bread and baked goods	0.721	1.84	21.964	21.964	0.959	2.44	22.83	22.83
Meat and meat products	0.561	1.44	17.082	39.045	1.049	2.67	24.988	47.818
Composite dishes	0.651	1.66	19.84	58.885	0.841	2.14	20.022	67.84
Sauces and dips	0.409	1.04	12.474	71.359	0.377	0.96	8.986	76.826
All sorts of cheese	0.308	0.78	9.372	80.731	0.29	0.74	6.907	83.732
Soups and bouillons	0.126	0.32	3.83	84.561	0.162	0.41	3.846	87.578
Snack/appetizer	0.099	0.25	3.016	87.577	0.104	0.26	2.475	90.053
Milk and milk products	0.1	0.25	3.043	90.62	0.103	0.26	2.443	92.496
Potatoes and potato products	0.089	0.23	2.717	93.337	0.111	0.28	2.648	95.144
Fish and seafood	0.097	0.25	2.946	96.283	0.097	0.25	2.304	97.449
Fruit and vegetables	0.07	0.18	2.126	98.409	0.053	0.13	1.26	98.709
Meat replacement products	0.031	0.08	0.957	99.366	0.025	0.06	0.604	99.313
Processed and unprocessed cereals	0.016	0.04	0.481	99.847	0.023	0.06	0.542	99.855
Coffee and tea	0.003	0.01	0.105	99.952	0.004	0.01	0.084	99.938
Fat spreads	0.002	0.00	0.048	100	0.003	0.01	0.062	100
**All foods** (w/o discretionary salt)	**3.3**	**8.3**			**4.2**	**10.7**		

**Use of discretionary salt**†	**n**	**%**	**n**	**%**
Yes	42	60.0%	30	42.3%
No	28	40.0%	41	57.7%

**Use of discretionary salt per salt intake estimate subgroup**‡	**n/N**	**%**	**n/N**	**%**
< 5.0 g/day	3/5	60.0%	1/2	50.0%
5.0-8.0 g/day§	12/27	44.4%	3/10	30.0%
> 8.0-10.0 g/day	13/20	65.0%	8/16	50.0%
> 10 g/day	14/18	77.8%	18/43	41.9%

* Assessed via semiquantitative sodium- and potassium-specific food record checklist (FRCL) on three consecutive days; does not include discretionary salt intake. [28]

† Participants reported having used salt/salt-containing condiments during at least one meal on one of three days.

‡ How to read (example): 3 out of 5 (60%) women with an estimated salt intake of less than 5 g/day reported having used discretionary salt/salt containing condiments.

§ Intermediate target range of Swiss salt reduction strategy. [4]

Supplementary Table S7 Frequency of classification of individual daily Na intake estimates in the same, adjacent, or opposite tertiles for test methods and reference method***** (N=141)

		PM-spot			FRCL		
	**Tertile**	**lower**	**middle**	**upper**	**lower**	**middle**	**upper**
**24hU**	**lower**	23 (16%)	18 (13%)	6 (4.3%)	25 (18%)	18 (13%)	4 (2.8%)
	**middle**	19 (13%)	18 (13%)	10 (7.1%)	17 (12%)	14 (9.9%)	16 (11%)
	**upper**	5 (3.5%)	11 (7.8%)	31 (22%)	5 (3.5%)	15 (11%)	27 (19%)

***** 24hU: Na intake calculated from excretion in 24-hour urine (reference method); PM-spot: Na intake predicted from excretion in late afternoon spot-urine, prediction model by Toft et al. [(12)]; FRCL, food record checklist [(28)]: questionnaire assessment of Na intake

Supplementary Table S8 Linear regression model to predict daily Na intake from FRCL adjusted by discretionary salt use*, women and men combined (N=141)

**Variable**	**Estimate**	**95% CI**	**p-value**
Intercept	0.459	[-0.606, 1.524]	0.395
Na (g/day) FRCL	0.856	[0.574, 1.138]	<0.001
Discretionary salt use (yes)	1.352	[-0.072, 2.777]	0.063
Na (g/day) FRCL : discretionary salt use (interaction)	-0.431	[-0.797, -0.064]	0.022
R^2 (adjusted): 0.2481			

***** FRCL, food record checklist [(28)]: questionnaire assessment of Na intake without accounting for discretionary salt intake; additional qualitative report about use of salt/salt-containing condiments during at least one meal on one of three days.

Figure S1 Daily Na intake estimates across methods, overall and by sex*

a Overall

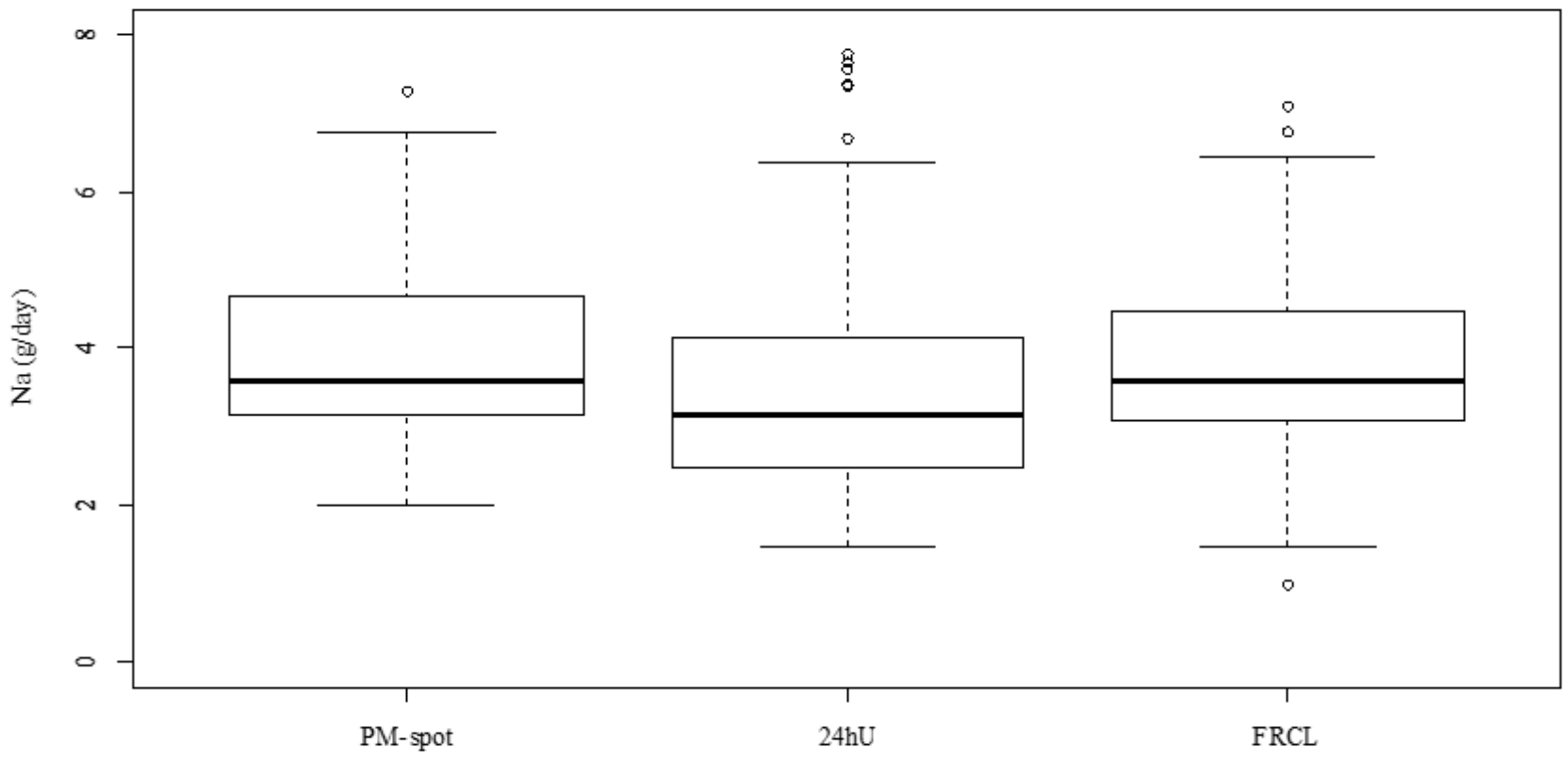

b Women

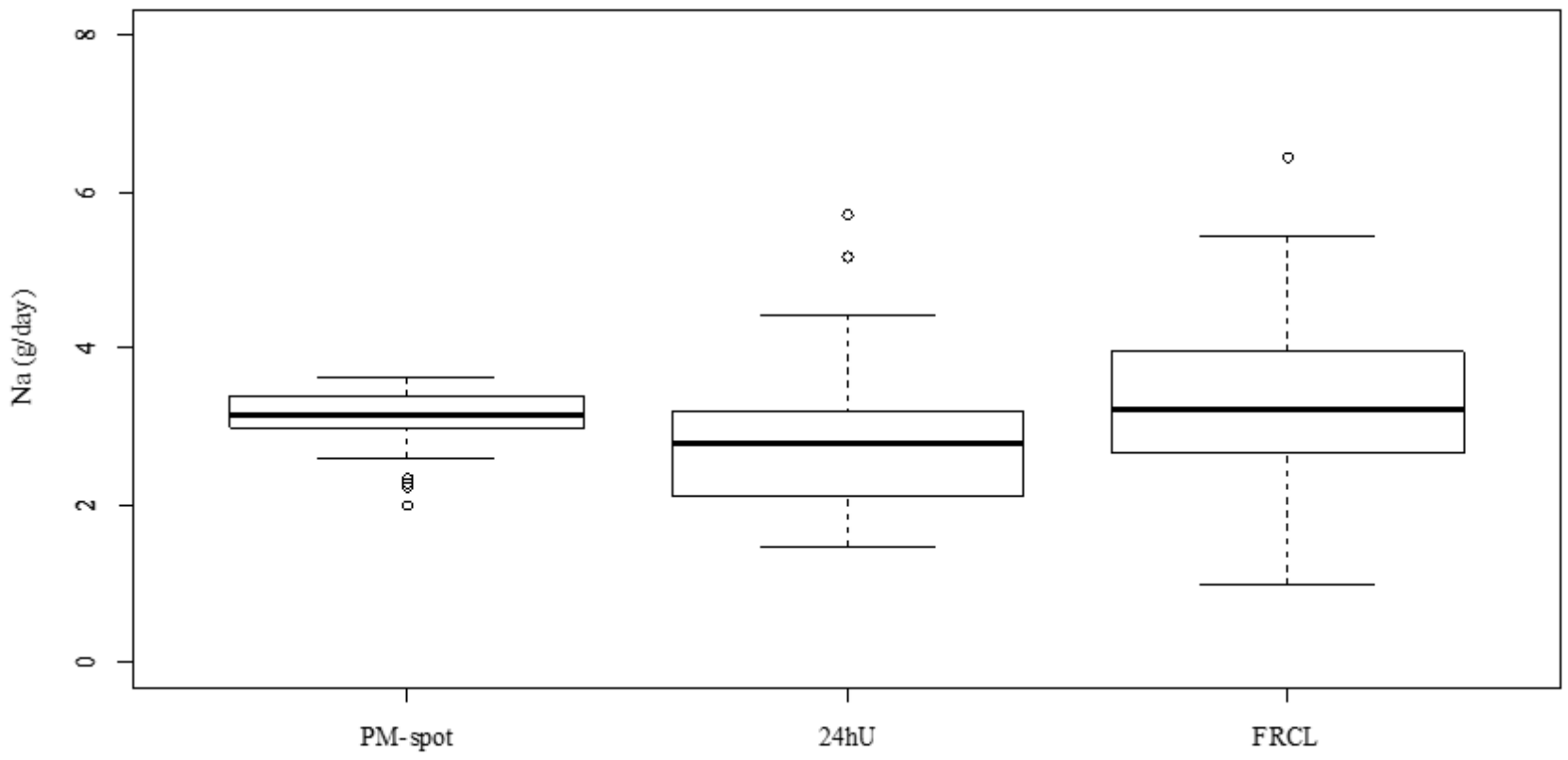

c Men

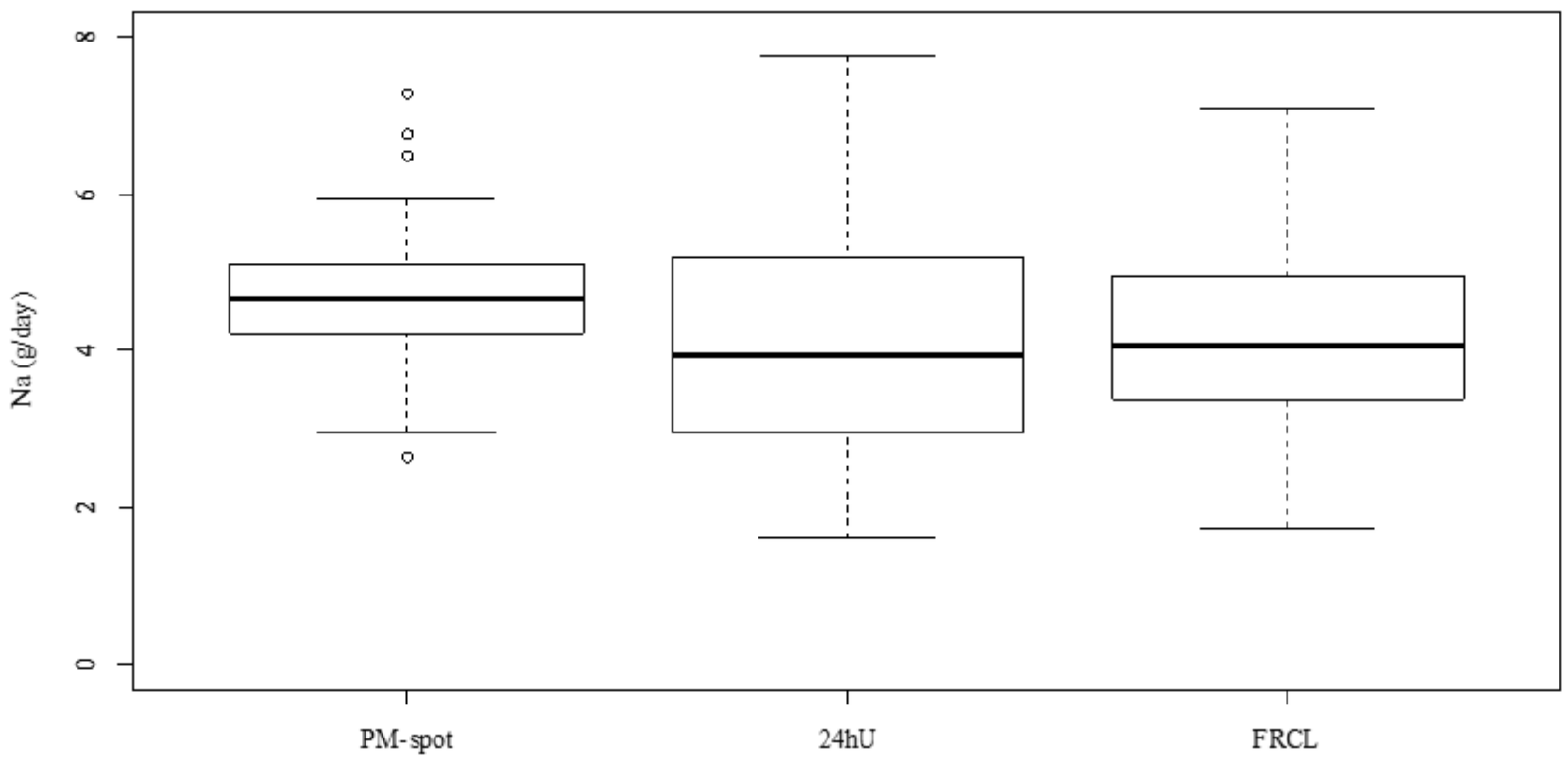

*Box plots of daily Na intake estimates from late afternoon spot-urine excretion (PM-spot) (Toft prediction model [12]), 24-hour urinary excretion (24hU), and assessment via food record checklist (FRCL) [28]; panel a, overall (N=141); panel b, in women (n=70); and panel c, in men (n=71)

Figure S2 Vision of a national salt intake monitoring system

Year 1	Year 2	Year 3	Year 4	Year 5	Year 6	Year 7	Year 8	Year 9	Year 10+
cover all seasons		cover all seasons	cover all seasons		cover all seasons	cover all seasons		Introduction of strategic measures for the next 8 years<br><br>Update of food composition database, tools etc.	cover all seasons
**Start 8-year period calibration point***		**Intermediate control point**	**Intermediate calibration* check point**		**Intermediate control point**	**End 8-year period recalibration* point**			**Intermediate control point**
Complete cohort<br>FRCL*<br>PM-spot*		Complete cohort<br>FRCL<br>PM-spot	Complete cohort<br>FRCL*<br>PM-spot*		Complete cohort<br>FRCL<br>PM-spot	Complete cohort<br>FRCL*<br>PM-spot*			Complete cohort<br>FRCL<br>PM-spot
Complete cohort<br>24hU		-----<br>-----	Cohort subsample<br>24hU		-----<br>-----	Complete cohort<br>24hU			-----<br>-----

*Repeated tests of the chosen, e.g., population-specific, prediction formulas of 24-hour Na excretion/intake; FRCL (food record checklist) and PM-spot (late afternoon spot-urine collection) compared with 24hU (24-hour urine collection).

Annotation for an application of the monitoring system in Switzerland as example:

The monitoring system evaluates salt reduction measures implemented in Switzerland (https://www.blv.admin.ch/blv/de/home/lebensmittel-und-ernaehrung/ernaehrung/produktzusammensetzung/salzstrategie.html). The eight-year monitoring period matches the Swiss nutrition strategy period (two legislative periods) and is of sufficient duration to detect changes and trends in population salt intake. The burden on the cohort is reduced by limiting 24-hour urine collection (24hU) to three 24hUs in eight years, and by only including a random subsample of the cohort at the midpoint. We expect the likelihood of dietary changes at time of 24hU will be low. More robust results might result from multiple individual 24hUs (calibration points), though at the risk of dropouts or reduced adherence to the collection protocol.
